# Two coordination modes of Cu^II^ in a binuclear complex with *N*-(pyridin-2-yl­carbon­yl)pyridine-2-carboxamidate ligands

**DOI:** 10.1107/S1600536812038330

**Published:** 2012-09-19

**Authors:** José J. Campos-Gaxiola, David Morales-Morales, Herbert Höpfl, Miguel Parra-Hake, Reyna Reyes-Martínez

**Affiliations:** a Facultad de Ingeniería Mochis, Universidad Autónoma de Sinaloa, Fuente de Poseidón y Prol. Ángel Flores, 81223 Los Mochis, Sinaloa, México; b Instituto de Química, Universidad Nacional Autónoma de México, Circuito exterior, Ciudad Universitaria, México, D.F., 04510, México; c Centro de Investigaciones Químicas, Universidad Autónoma del estado de Morelos, Av. Universidad 1001, 62209 Cuernavaca, Morelos, México; d Centro de Graduados e Investigación del Instituto Tecnológico de Tijuana, Apdo. Postal 1166, 22500 Tijuana, BC, México

## Abstract

In the title dinuclear complex, (acetonitrile-1κ*N*)[μ-*N*-(pyri­din-2-ylcarbonyl)pyridine-2-carboxamidato-1:2κ^5^ *N*,*N*′,*N*′′:*O*,*O*′][*N*-(pyridin-2-ylcarbonyl)pyridine-2-carboxamidato-2κ^3^ *N*,*N*′,*N*′′]bis(trifluoromethanesulfonato-1κ*O*)dicopper(II), [Cu_2_(C_12_H_8_N_3_O_2_)_2_(CF_3_O_3_S)_2_(CH_3_CN)], one of the Cu^II^ ions is five-coordinated in a distorted square-pyramidal N_3_O_2_ environment provided by two *N*-(pyridin-2-ylcarbon­yl)pyridine-2-carboxamidate (bpca) ligands, while the second Cu^II^ ion is six-coordinated in a distorted octa­hedral N_4_O_2_ environment provided by one bpca ligand, two trifluoro­methansulfonate ligands and one acetonitrile mol­ecule. Weak inter­molecular C—H⋯O and C—H⋯F hydrogen bonds and π–π stacking inter­actions with centroid–centroid distances of 3.6799 (15) and 3.8520 (16) Å stabilize the crystal packing and lead to a three-dimensional network.

## Related literature

For complexes of divalent metal ions with the *N*-(pyridin-2-ylcarbon­yl)pyridine-2-carboxamidate (bpca) ligand, see: Chowdhury *et al.* (2007 ▶); Folgado *et al.* (1988 ▶); Ha (2010 ▶, 2011 ▶); Halder *et al.* (2010 ▶); Miguel *et al.* (2009 ▶). For complexes of trivalent metal ions with the bpca ligand, see: Li *et al.* (2011 ▶); Sugimoto *et al.* (2002 ▶); Wocadlo *et al.* (1993 ▶). For electrochemical and magnetic studies for example complexes of Cu(II), see: Cangussu de Castro Gomes *et al.* (2008 ▶); Kajiwara *et al.* (2002 ▶). For the synthesis of the ligand, see: Larter *et al.* (1998 ▶).
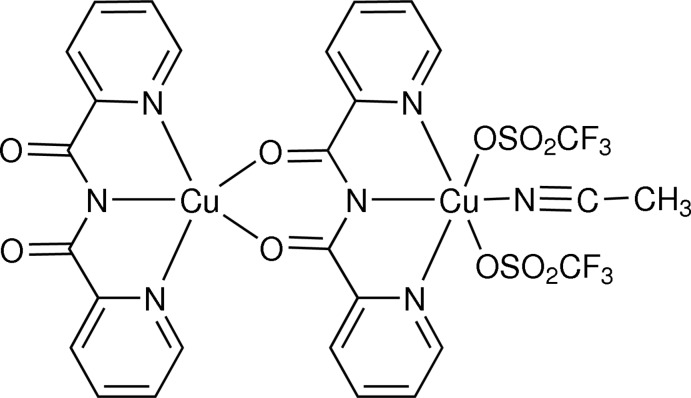


## Experimental

### 

#### Crystal data

[Cu_2_(C_12_H_8_N_3_O_2_)_2_(CF_3_O_3_S)_2_(C_2_H_3_N)]
*M* *_r_* = 918.70
Triclinic, 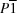

*a* = 8.9726 (7) Å
*b* = 10.0569 (8) Å
*c* = 18.3689 (15) Å
α = 82.573 (1)°
β = 83.802 (1)°
γ = 82.222 (1)°
*V* = 1621.6 (2) Å^3^
*Z* = 2
Mo *K*α radiation
μ = 1.55 mm^−1^
*T* = 100 K
0.32 × 0.24 × 0.18 mm

#### Data collection

Bruker SMART CCD area-detector diffractometer
Absorption correction: multi-scan (*SADABS*; Bruker, 2001 ▶) *T*_min_ = 0.638, *T*_max_ = 0.768
15254 measured reflections
5686 independent reflections
5292 reflections with *I* > 2σ(*I*)
*R*_int_ = 0.024

#### Refinement

*R*[*F* ^2^ > 2σ(*F* ^2^)] = 0.033
*wR*(*F* ^2^) = 0.087
*S* = 1.04
5686 reflections
497 parameters
H-atom parameters constrained
Δρ_max_ = 0.83 e Å^−3^
Δρ_min_ = −0.50 e Å^−3^

### 

Data collection: *SMART* (Bruker, 2001 ▶); cell refinement: *SAINT-Plus* (Bruker, 2001 ▶); data reduction: *SAINT-Plus*; program(s) used to solve structure: *SHELXS97* (Sheldrick, 2008 ▶); program(s) used to refine structure: *SHELXL97* (Sheldrick, 2008 ▶); molecular graphics: *SHELXTL* (Sheldrick, 2008 ▶) and *DIAMOND* (Brandenburg, 2006 ▶); software used to prepare material for publication: *SHELXTL* and *PLATON* (Spek, 2009 ▶).

## Supplementary Material

Crystal structure: contains datablock(s) I, global. DOI: 10.1107/S1600536812038330/wm2669sup1.cif

Structure factors: contains datablock(s) I. DOI: 10.1107/S1600536812038330/wm2669Isup2.hkl

Additional supplementary materials:  crystallographic information; 3D view; checkCIF report

## Figures and Tables

**Table 1:** Hydrogen-bond geometry (Å, °)

*D*—H⋯*A*	*D*—H	H⋯*A*	*D*⋯*A*	*D*—H⋯*A*
C2—H2⋯O9^i^	0.95	2.57	3.448 (3)	154
C4—H4⋯O1^ii^	0.95	2.53	3.210 (4)	128
C10—H10⋯F5^iii^	0.95	2.54	3.239 (3)	130
C21—H21⋯O1^iv^	0.95	2.48	3.257 (3)	140
C21—H21⋯O2^iv^	0.95	2.37	3.203 (3)	147
C22—H22⋯O10^v^	0.95	2.48	3.400 (3)	163

Symmetry codes: (i) 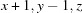; (ii) 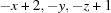; (iii) 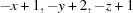; (iv) 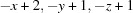; (v) 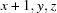.

**Fig. 1.**
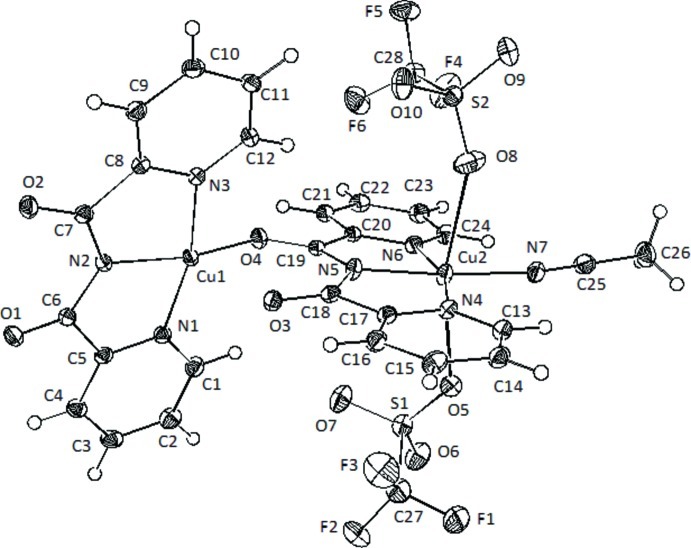
The molecular structure of the title compound with displacement ellipsoids at the 40% probability level.

**Fig. 2.**
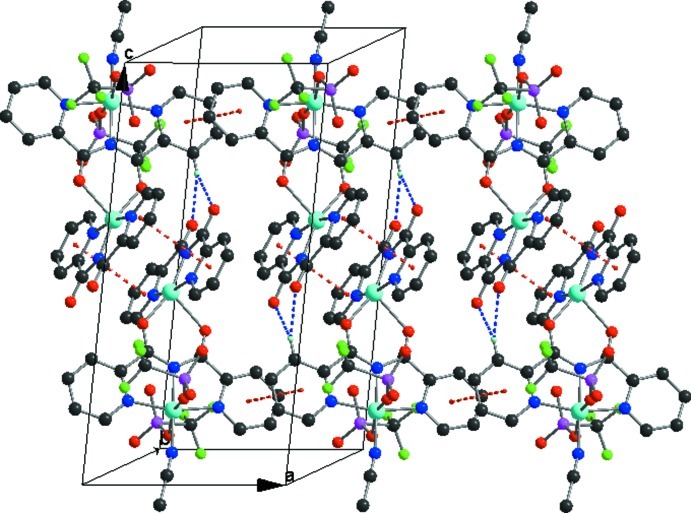
A part of the crystal packing of the title compound, showing intermolecular C—H···O and π···π interactions. Hydrogen atoms not involved in hydrogen bonding have been omitted for clarity.

## supplementary crystallographic information

### Comment

N-(pyridin-2-ylcarbonyl)pyridine-2-carboxamidate (bpca) is a rigid tridentate ligand that can act as a bridging ligand to produce supramolecular structures based on organic-inorganic coordination frameworks (Halder *et al.*, 2010). This ligand exhibits various coordination modes. It can also appear as a regular tridentate chelating ligand *via* its two pyridine and one amine N atoms, or in a bidentate manner through the carboxyl groups. The bpca ligand forms coordination complexes with a large number of divalent metal ions, e.g. Pd(II) (Ha, 2010; Miguel *et al.*, 2009), Pt(II) (Ha, 2011), Cu(II) (Folgado *et al.*, 1988; Chowdhury *et al.*, 2007), or with trivalent metals ions, e.g. Fe(III) (Wocadlo *et al.*, 1993; Li *et al.*, 2011), Cr(III) (Kajiwara *et al.*, 2002), Co(III), Re(III) (Sugimoto *et al.*, 2002)]. Mono and multinuclear complexes with bpca have been the subject of various electrochemical and magnetic studies, for example of complexes of Cu(II) (Cangussu de Castro Gomes *et al.*, 2008; Chowdhury *et al.*, 2007) and Co(III) (Kajiwara *et al.*, 2002). In this context we report here the crystal structure for the dinuclear copper complex [Cu_2_(C_12_H_8_N_3_O_2_)_2_(CF_3_SO_3_)_2_(CH_3_CN)], or [(bpca)Cu(µ-bpca)Cu(OSO_2_CF_3_)_2_(NCCH_3_)]. An ORTEP-style plot of the molecular structure including the atom numbering is shown in Figure 1.

The above mentioned complex presents the copper(II) ion Cu1 in a five-coordinate environment in a somewhat distorted square pyramidal geometry. The coordination includes two bpca ligands. One ligand acts as a tridentate *N,N'N''*-chelate through two pyridine nitrogen atoms (Cu—N1 = 1.978 (2) and Cu—N3 = 1.988 (2) Å) and one amide nitrogen atom in basal positions (Cu—N2 = 1.922 (2) Å); the forth basal position is occupied by one carbonyl O atom (O4) from the bridging ligand with a Cu—O distance of 1.977 (18) while the second carbonyl O atom (O3) is at the apical site at a distance of 2.2452 (18). The six-coordinate Cu2 ion is found in a distorted octahedral geometry, defined by the N atoms of the bridging bpca ligand and one acetonitrile molecule in equatorial positions and two trifluoromethansulfonate O atoms with distances Cu—O of 2.439 (2) and 2.482 (2) Å in axial positions. The angles between the copper atoms and the bpca ligands vary in the range of 81.49 (9) - 82.83 (9) ° evidencing the small bite angle of the corresponding five membered chelate rings.

In the crystal lattice, the dinuclear units are packed through intermolecular C—H···*A* (*A* = O, F) hydrogen bonds (Table 1) between the pyridine ring hydrogen atoms and the carbonyl oxygen atoms (O1, O2) of the bpca ligand, the sulfonyl oxygen (O9, O10) and the fluor atom (F5) of the trifluoromethansulfonate ligands. The pyridine rings interact also *via*π–π stacking interactions, with *Cg*—*Cg* distances of 3.6799 (15) Å for the interaction between the tridentate bpca ligands, and of 3.8520 (16) Å for the interactions between the bridging bpca ligands (Fig. 2).

### Experimental

The starting material *trans*-(±)-2,4,5-tris(pyridine-2-yl)-2-imidazoline was synthesized according to a previously reported procedure (Larter *et al.*, 1998). For the preparation of the title compound, a mixture of *trans*-(±)-2,4,5-tris(pyridine-2-yl)-2-imidazoline (0.086 g, 0.1382 mmol) and Cu(OSO_2_CF_3_)_2_ (0.050 g, 0.1382 mmol) was dissolved in acetonitrile (5 ml) and stirred for 24 h at room temperature to afford a green solution. The product was crystallized at room temperature by gas phase diffusion of diethyl ether into the reaction mixture, producing green crystals which were separated and further dried under vacuum. Yield: 24%. IR (KBr): 3082, 3030, 1707, 1672, 1626, 1587, 1544, 1462, 1380, 1323, 1264, 1239, 1144, 1013, 740 cm^-1^. MS [FAB^+^, m/*z* (%)]: 729 (2) [(*M*+H)-OSO_2_CF_3_—NCCH_3_]^+^.

### Refinement

H atoms were included in calculated positions (C—H = 0.95 Å for aromatic H, C—H=0.98 Å for methyl H), and refined using a riding model, with *U*_iso_(H) = 1.2*U*_eq_ and *U*_iso_(H) = 1.5*U*_eq_ for methyl H atoms.

### Crystal data

**Table:**

[Cu_2_(C_12_H_8_N_3_O_2_)_2_(CF_3_SO_3_)_2_(C_2_H_3_N)]	*Z* = 2
*M**_r_* = 918.70	*F*(000) = 920
Triclinic, *P*1	*D*_x_ = 1.882 Mg m^−^^3^
Hall symbol: -P 1	Mo *K*α radiation, λ = 0.71073 Å
*a* = 8.9726 (7) Å	Cell parameters from 6358 reflections
*b* = 10.0569 (8) Å	θ = 2.2–28.3°
*c* = 18.3689 (15) Å	µ = 1.55 mm^−^^1^
α = 82.573 (1)°	*T* = 100 K
β = 83.802 (1)°	Rectangular prism, green
γ = 82.222 (1)°	0.32 × 0.24 × 0.18 mm
*V* = 1621.6 (2) Å^3^	

### Data collection

**Table:**

Bruker SMART CCD area-detector diffractometer	5686 independent reflections
Radiation source: fine-focus sealed tube	5292 reflections with *I* > 2σ(*I*)
Graphite monochromator	*R*_int_ = 0.024
phi and ω scans	θ_max_ = 25.0°, θ_min_ = 2.1°
Absorption correction: multi-scan (*SADABS*; Bruker, 2001)	*h* = −10→10
*T*_min_ = 0.638, *T*_max_ = 0.768	*k* = −11→11
15254 measured reflections	*l* = −21→21

### Refinement

**Table:**

Refinement on *F*^2^	Primary atom site location: structure-invariant direct methods
Least-squares matrix: full	Secondary atom site location: difference Fourier map
*R*[*F*^2^ > 2σ(*F*^2^)] = 0.033	Hydrogen site location: inferred from neighbouring sites
*wR*(*F*^2^) = 0.087	H-atom parameters constrained
*S* = 1.04	*w* = 1/[σ^2^(*F*_o_^2^) + (0.0451*P*)^2^ + 2.0646*P*] where *P* = (*F*_o_^2^ + 2*F*_c_^2^)/3
5686 reflections	(Δ/σ)_max_ = 0.008
497 parameters	Δρ_max_ = 0.83 e Å^−^^3^
0 restraints	Δρ_min_ = −0.50 e Å^−^^3^

### Special details

**Table:**

Geometry. All e.s.d.'s (except the e.s.d. in the dihedral angle between two l.s. planes) are estimated using the full covariance matrix. The cell e.s.d.'s are taken into account individually in the estimation of e.s.d.'s in distances, angles and torsion angles; correlations between e.s.d.'s in cell parameters are only used when they are defined by crystal symmetry. An approximate (isotropic) treatment of cell e.s.d.'s is used for estimating e.s.d.'s involving l.s. planes.
Refinement. Refinement of *F*^2^ against ALL reflections. The weighted *R*-factor *wR* and goodness of fit *S* are based on *F*^2^, conventional *R*-factors *R* are based on *F*, with *F* set to zero for negative *F*^2^. The threshold expression of *F*^2^ > σ(*F*^2^) is used only for calculating *R*-factors(gt) *etc*. and is not relevant to the choice of reflections for refinement. *R*-factors based on *F*^2^ are statistically about twice as large as those based on *F*, and *R*- factors based on ALL data will be even larger.

### Fractional atomic coordinates and isotropic or equivalent isotropic displacement parameters (Å^2^)

**Table:**

	*x*	*y*	*z*	*U*_iso_*/*U*_eq_	
Cu1	0.84461 (3)	0.49089 (3)	0.586455 (16)	0.01596 (10)	
Cu2	0.71274 (4)	0.65971 (3)	0.851929 (17)	0.02035 (10)	
S1	0.90238 (8)	0.33964 (7)	0.90897 (4)	0.02539 (16)	
S2	0.55073 (7)	0.99281 (7)	0.74959 (4)	0.02062 (15)	
F1	0.7268 (3)	0.1888 (2)	0.99513 (11)	0.0517 (6)	
F2	0.8668 (3)	0.08502 (19)	0.91195 (12)	0.0532 (6)	
F3	0.6749 (2)	0.2241 (2)	0.88165 (13)	0.0564 (6)	
F4	0.8208 (2)	1.0523 (2)	0.76493 (11)	0.0508 (5)	
F5	0.7233 (2)	1.15490 (17)	0.66873 (11)	0.0381 (4)	
F6	0.8107 (2)	0.94594 (19)	0.67342 (11)	0.0416 (5)	
N1	0.9880 (2)	0.3263 (2)	0.60772 (11)	0.0176 (4)	
N2	0.8212 (2)	0.4073 (2)	0.50069 (11)	0.0170 (4)	
N3	0.6971 (2)	0.6358 (2)	0.54179 (11)	0.0175 (4)	
N4	0.5243 (2)	0.5723 (2)	0.85181 (12)	0.0197 (5)	
N5	0.7535 (2)	0.5968 (2)	0.75566 (12)	0.0201 (5)	
N6	0.9188 (2)	0.7201 (2)	0.82471 (12)	0.0191 (5)	
N7	0.6692 (3)	0.7400 (2)	0.94561 (12)	0.0237 (5)	
O1	0.8791 (2)	0.20122 (18)	0.45319 (10)	0.0241 (4)	
O2	0.6910 (2)	0.44173 (19)	0.39529 (10)	0.0236 (4)	
O3	0.6675 (2)	0.45898 (18)	0.68097 (9)	0.0202 (4)	
O4	0.9287 (2)	0.59552 (18)	0.65287 (10)	0.0200 (4)	
O5	0.7980 (2)	0.4528 (2)	0.92880 (11)	0.0309 (5)	
O6	1.0187 (2)	0.2967 (2)	0.95778 (12)	0.0374 (5)	
O7	0.9513 (2)	0.3449 (2)	0.83152 (11)	0.0367 (5)	
O8	0.5841 (3)	0.8840 (2)	0.80548 (13)	0.0442 (6)	
O9	0.4725 (2)	1.1139 (2)	0.77653 (12)	0.0339 (5)	
O10	0.4920 (2)	0.9585 (2)	0.68607 (12)	0.0342 (5)	
C1	1.0716 (3)	0.2935 (3)	0.66516 (15)	0.0228 (6)	
H1	1.0666	0.3552	0.7006	0.027*	
C2	1.1649 (3)	0.1725 (3)	0.67427 (16)	0.0264 (6)	
H2	1.2239	0.1514	0.7152	0.032*	
C3	1.1708 (3)	0.0831 (3)	0.62301 (15)	0.0246 (6)	
H3	1.2344	−0.0005	0.6281	0.030*	
C4	1.0842 (3)	0.1154 (3)	0.56420 (15)	0.0211 (6)	
H4	1.0867	0.0546	0.5284	0.025*	
C5	0.9936 (3)	0.2380 (3)	0.55840 (14)	0.0176 (5)	
C6	0.8920 (3)	0.2789 (2)	0.49648 (14)	0.0175 (5)	
C7	0.7229 (3)	0.4747 (3)	0.45235 (14)	0.0181 (5)	
C8	0.6495 (3)	0.6052 (3)	0.47920 (13)	0.0171 (5)	
C9	0.5377 (3)	0.6875 (3)	0.44324 (14)	0.0201 (5)	
H9	0.5069	0.6650	0.3989	0.024*	
C10	0.4715 (3)	0.8028 (3)	0.47282 (15)	0.0228 (6)	
H10	0.3949	0.8616	0.4489	0.027*	
C11	0.5179 (3)	0.8319 (3)	0.53755 (15)	0.0223 (6)	
H11	0.4716	0.9097	0.5594	0.027*	
C12	0.6315 (3)	0.7474 (3)	0.57013 (14)	0.0207 (6)	
H12	0.6645	0.7690	0.6141	0.025*	
C13	0.4100 (3)	0.5647 (3)	0.90416 (15)	0.0232 (6)	
H13	0.4128	0.6060	0.9476	0.028*	
C14	0.2871 (3)	0.4986 (3)	0.89746 (15)	0.0243 (6)	
H14	0.2068	0.4951	0.9356	0.029*	
C15	0.2829 (3)	0.4384 (3)	0.83506 (15)	0.0258 (6)	
H15	0.1990	0.3937	0.8291	0.031*	
C16	0.4027 (3)	0.4434 (3)	0.78055 (15)	0.0230 (6)	
H16	0.4031	0.4012	0.7371	0.028*	
C17	0.5205 (3)	0.5109 (3)	0.79105 (14)	0.0187 (5)	
C18	0.6545 (3)	0.5187 (3)	0.73564 (14)	0.0187 (5)	
C19	0.8827 (3)	0.6220 (2)	0.71644 (13)	0.0166 (5)	
C20	0.9778 (3)	0.6941 (2)	0.75634 (14)	0.0176 (5)	
C21	1.1151 (3)	0.7301 (3)	0.72523 (14)	0.0201 (5)	
H21	1.1531	0.7101	0.6770	0.024*	
C22	1.1966 (3)	0.7964 (3)	0.76622 (15)	0.0232 (6)	
H22	1.2918	0.8230	0.7466	0.028*	
C23	1.1370 (3)	0.8229 (3)	0.83589 (15)	0.0252 (6)	
H23	1.1909	0.8681	0.8649	0.030*	
C24	0.9979 (3)	0.7832 (3)	0.86351 (15)	0.0233 (6)	
H24	0.9579	0.8018	0.9117	0.028*	
C25	0.6605 (3)	0.7991 (3)	0.99484 (16)	0.0266 (6)	
C26	0.6546 (4)	0.8740 (3)	1.05813 (17)	0.0329 (7)	
H26A	0.7570	0.8726	1.0724	0.049*	
H26B	0.5919	0.8321	1.0995	0.049*	
H26C	0.6110	0.9677	1.0451	0.049*	
C27	0.7879 (4)	0.2023 (3)	0.92485 (18)	0.0374 (7)	
C28	0.7356 (3)	1.0402 (3)	0.71278 (16)	0.0254 (6)	

### Atomic displacement parameters (Å^2^)

**Table:**

	*U*^11^	*U*^22^	*U*^33^	*U*^12^	*U*^13^	*U*^23^
Cu1	0.01840 (17)	0.01472 (17)	0.01487 (17)	−0.00005 (12)	−0.00226 (12)	−0.00360 (12)
Cu2	0.02125 (18)	0.02642 (19)	0.01445 (17)	−0.00578 (14)	−0.00002 (12)	−0.00476 (13)
S1	0.0219 (3)	0.0308 (4)	0.0235 (4)	−0.0026 (3)	−0.0030 (3)	−0.0034 (3)
S2	0.0196 (3)	0.0222 (3)	0.0200 (3)	−0.0035 (3)	−0.0015 (3)	−0.0011 (3)
F1	0.0657 (14)	0.0437 (12)	0.0441 (12)	−0.0192 (10)	0.0183 (10)	−0.0058 (9)
F2	0.0772 (16)	0.0270 (10)	0.0535 (13)	0.0020 (10)	−0.0004 (11)	−0.0113 (9)
F3	0.0441 (12)	0.0621 (14)	0.0711 (15)	−0.0188 (11)	−0.0180 (11)	−0.0151 (12)
F4	0.0324 (11)	0.0827 (16)	0.0427 (11)	−0.0211 (10)	−0.0145 (9)	−0.0035 (11)
F5	0.0342 (10)	0.0265 (9)	0.0497 (11)	−0.0080 (8)	0.0021 (8)	0.0093 (8)
F6	0.0282 (10)	0.0393 (11)	0.0543 (12)	−0.0005 (8)	0.0117 (8)	−0.0110 (9)
N1	0.0170 (11)	0.0177 (11)	0.0173 (11)	−0.0013 (9)	−0.0005 (8)	−0.0009 (9)
N2	0.0201 (11)	0.0147 (11)	0.0160 (10)	0.0000 (9)	−0.0023 (8)	−0.0034 (8)
N3	0.0207 (11)	0.0159 (11)	0.0153 (10)	−0.0013 (9)	0.0000 (8)	−0.0012 (8)
N4	0.0212 (11)	0.0215 (11)	0.0160 (11)	−0.0038 (9)	−0.0004 (9)	−0.0003 (9)
N5	0.0198 (11)	0.0267 (12)	0.0150 (11)	−0.0057 (9)	0.0001 (9)	−0.0051 (9)
N6	0.0200 (11)	0.0203 (11)	0.0168 (11)	−0.0011 (9)	−0.0036 (9)	−0.0017 (9)
N7	0.0284 (13)	0.0250 (12)	0.0177 (12)	−0.0045 (10)	0.0005 (9)	−0.0036 (10)
O1	0.0318 (11)	0.0180 (9)	0.0230 (10)	0.0017 (8)	−0.0063 (8)	−0.0066 (8)
O2	0.0283 (10)	0.0242 (10)	0.0186 (10)	0.0030 (8)	−0.0063 (8)	−0.0070 (8)
O3	0.0218 (9)	0.0218 (9)	0.0180 (9)	−0.0059 (8)	0.0003 (7)	−0.0037 (8)
O4	0.0204 (9)	0.0214 (9)	0.0194 (9)	−0.0044 (7)	0.0000 (7)	−0.0066 (7)
O5	0.0397 (12)	0.0265 (11)	0.0255 (11)	−0.0026 (9)	−0.0018 (9)	−0.0025 (8)
O6	0.0278 (11)	0.0557 (15)	0.0284 (11)	−0.0046 (10)	−0.0080 (9)	0.0005 (10)
O7	0.0315 (12)	0.0515 (14)	0.0247 (11)	−0.0044 (10)	0.0033 (9)	−0.0016 (10)
O8	0.0366 (13)	0.0437 (14)	0.0445 (14)	−0.0016 (11)	−0.0006 (10)	0.0184 (11)
O9	0.0334 (12)	0.0347 (12)	0.0344 (12)	−0.0005 (9)	0.0019 (9)	−0.0150 (9)
O10	0.0265 (11)	0.0491 (13)	0.0312 (11)	−0.0105 (10)	0.0000 (9)	−0.0169 (10)
C1	0.0234 (14)	0.0253 (14)	0.0195 (13)	−0.0017 (11)	−0.0030 (11)	−0.0023 (11)
C2	0.0222 (14)	0.0305 (16)	0.0252 (14)	0.0008 (12)	−0.0060 (11)	0.0009 (12)
C3	0.0209 (14)	0.0205 (14)	0.0289 (15)	0.0035 (11)	−0.0001 (11)	0.0024 (11)
C4	0.0194 (13)	0.0186 (13)	0.0242 (14)	−0.0009 (10)	0.0007 (11)	−0.0022 (11)
C5	0.0172 (13)	0.0169 (12)	0.0178 (13)	−0.0043 (10)	0.0023 (10)	0.0006 (10)
C6	0.0190 (13)	0.0161 (13)	0.0167 (12)	−0.0018 (10)	0.0008 (10)	−0.0017 (10)
C7	0.0180 (13)	0.0180 (13)	0.0171 (13)	−0.0025 (10)	0.0018 (10)	−0.0004 (10)
C8	0.0194 (13)	0.0164 (13)	0.0150 (12)	−0.0027 (10)	−0.0003 (10)	−0.0005 (10)
C9	0.0206 (13)	0.0205 (13)	0.0183 (13)	−0.0024 (11)	−0.0005 (10)	−0.0002 (10)
C10	0.0219 (14)	0.0209 (14)	0.0224 (14)	0.0013 (11)	0.0005 (11)	0.0027 (11)
C11	0.0262 (14)	0.0150 (13)	0.0229 (14)	0.0010 (11)	0.0046 (11)	−0.0013 (10)
C12	0.0267 (14)	0.0173 (13)	0.0182 (13)	−0.0008 (11)	−0.0017 (11)	−0.0047 (10)
C13	0.0265 (14)	0.0235 (14)	0.0178 (13)	−0.0005 (11)	0.0014 (11)	−0.0011 (11)
C14	0.0231 (14)	0.0257 (14)	0.0215 (14)	−0.0013 (11)	0.0031 (11)	0.0012 (11)
C15	0.0226 (14)	0.0289 (15)	0.0258 (15)	−0.0089 (12)	0.0003 (11)	0.0017 (12)
C16	0.0258 (14)	0.0237 (14)	0.0196 (13)	−0.0045 (11)	−0.0020 (11)	−0.0015 (11)
C17	0.0203 (13)	0.0180 (13)	0.0169 (13)	−0.0009 (10)	−0.0016 (10)	0.0004 (10)
C18	0.0203 (13)	0.0182 (13)	0.0169 (13)	−0.0008 (10)	−0.0038 (10)	0.0010 (10)
C19	0.0197 (13)	0.0141 (12)	0.0152 (12)	0.0006 (10)	−0.0024 (10)	−0.0010 (10)
C20	0.0211 (13)	0.0147 (12)	0.0162 (12)	0.0015 (10)	−0.0040 (10)	−0.0007 (10)
C21	0.0212 (13)	0.0194 (13)	0.0192 (13)	−0.0004 (10)	−0.0031 (10)	−0.0019 (10)
C22	0.0208 (14)	0.0225 (14)	0.0266 (14)	−0.0054 (11)	−0.0036 (11)	0.0000 (11)
C23	0.0303 (15)	0.0233 (14)	0.0245 (14)	−0.0070 (12)	−0.0085 (12)	−0.0028 (11)
C24	0.0273 (15)	0.0250 (14)	0.0191 (13)	−0.0046 (11)	−0.0056 (11)	−0.0035 (11)
C25	0.0262 (15)	0.0247 (15)	0.0270 (16)	−0.0023 (12)	0.0002 (12)	0.0009 (13)
C26	0.0398 (18)	0.0337 (17)	0.0271 (16)	−0.0023 (14)	−0.0038 (13)	−0.0124 (13)
C27	0.0411 (19)	0.0339 (18)	0.0372 (18)	−0.0062 (14)	0.0011 (15)	−0.0063 (14)
C28	0.0231 (14)	0.0252 (15)	0.0277 (15)	−0.0035 (12)	−0.0039 (11)	−0.0002 (12)

### Geometric parameters (Å, º)

**Table:**

Cu1—N2	1.922 (2)	C1—C2	1.382 (4)
Cu1—O4	1.9772 (18)	C1—H1	0.9500
Cu1—N1	1.978 (2)	C2—C3	1.376 (4)
Cu1—N3	1.988 (2)	C2—H2	0.9500
Cu1—O3	2.2452 (18)	C3—C4	1.378 (4)
Cu2—N5	1.936 (2)	C3—H3	0.9500
Cu2—N7	1.973 (2)	C4—C5	1.381 (4)
Cu2—N4	2.008 (2)	C4—H4	0.9500
Cu2—N6	2.016 (2)	C5—C6	1.515 (4)
Cu2—O5	2.439 (2)	C7—C8	1.505 (3)
S1—O5	1.432 (2)	C8—C9	1.380 (4)
S1—O6	1.435 (2)	C9—C10	1.378 (4)
S1—O7	1.439 (2)	C9—H9	0.9500
S1—C27	1.807 (3)	C10—C11	1.379 (4)
S2—O8	1.424 (2)	C10—H10	0.9500
S2—O10	1.430 (2)	C11—C12	1.373 (4)
S2—O9	1.439 (2)	C11—H11	0.9500
S2—C28	1.823 (3)	C12—H12	0.9500
F1—C27	1.344 (4)	C13—C14	1.387 (4)
F2—C27	1.326 (4)	C13—H13	0.9500
F3—C27	1.332 (4)	C14—C15	1.370 (4)
F4—C28	1.316 (3)	C14—H14	0.9500
F5—C28	1.320 (3)	C15—C16	1.389 (4)
F6—C28	1.332 (3)	C15—H15	0.9500
N1—C5	1.341 (3)	C16—C17	1.374 (4)
N1—C1	1.341 (3)	C16—H16	0.9500
N2—C6	1.367 (3)	C17—C18	1.493 (4)
N2—C7	1.371 (3)	C19—C20	1.493 (4)
N3—C12	1.335 (3)	C20—C21	1.375 (4)
N3—C8	1.354 (3)	C21—C22	1.388 (4)
N4—C13	1.331 (4)	C21—H21	0.9500
N4—C17	1.349 (3)	C22—C23	1.377 (4)
N5—C19	1.333 (3)	C22—H22	0.9500
N5—C18	1.370 (3)	C23—C24	1.385 (4)
N6—C24	1.327 (3)	C23—H23	0.9500
N6—C20	1.353 (3)	C24—H24	0.9500
N7—C25	1.135 (4)	C25—C26	1.457 (4)
O1—C6	1.209 (3)	C26—H26A	0.9800
O2—C7	1.213 (3)	C26—H26B	0.9800
O3—C18	1.222 (3)	C26—H26C	0.9800
O4—C19	1.246 (3)		
			
N2—Cu1—O4	160.54 (8)	O2—C7—C8	120.4 (2)
N2—Cu1—N1	82.83 (9)	N2—C7—C8	110.6 (2)
O4—Cu1—N1	94.22 (8)	N3—C8—C9	121.9 (2)
N2—Cu1—N3	82.65 (9)	N3—C8—C7	116.0 (2)
O4—Cu1—N3	99.18 (8)	C9—C8—C7	122.1 (2)
N1—Cu1—N3	165.44 (9)	C10—C9—C8	118.7 (2)
N2—Cu1—O3	115.15 (8)	C10—C9—H9	120.6
O4—Cu1—O3	84.30 (7)	C8—C9—H9	120.6
N1—Cu1—O3	99.70 (8)	C9—C10—C11	119.2 (2)
N3—Cu1—O3	87.33 (8)	C9—C10—H10	120.4
N5—Cu2—N7	175.01 (10)	C11—C10—H10	120.4
N5—Cu2—N4	81.63 (9)	C12—C11—C10	119.4 (2)
N7—Cu2—N4	100.19 (9)	C12—C11—H11	120.3
N5—Cu2—N6	81.49 (9)	C10—C11—H11	120.3
N7—Cu2—N6	96.84 (9)	N3—C12—C11	122.0 (2)
N4—Cu2—N6	162.92 (9)	N3—C12—H12	119.0
N5—Cu2—O5	99.74 (8)	C11—C12—H12	119.0
N7—Cu2—O5	85.09 (8)	N4—C13—C14	122.3 (3)
N4—Cu2—O5	84.15 (8)	N4—C13—H13	118.9
N6—Cu2—O5	96.16 (8)	C14—C13—H13	118.9
O5—S1—O6	114.89 (13)	C15—C14—C13	119.1 (3)
O5—S1—O7	114.47 (13)	C15—C14—H14	120.5
O6—S1—O7	115.54 (13)	C13—C14—H14	120.5
O5—S1—C27	102.93 (14)	C14—C15—C16	119.3 (3)
O6—S1—C27	103.22 (15)	C14—C15—H15	120.3
O7—S1—C27	103.38 (15)	C16—C15—H15	120.3
O8—S2—O10	115.97 (15)	C17—C16—C15	118.3 (3)
O8—S2—O9	114.56 (14)	C17—C16—H16	120.9
O10—S2—O9	113.69 (13)	C15—C16—H16	120.9
O8—S2—C28	103.89 (13)	N4—C17—C16	122.8 (2)
O10—S2—C28	102.71 (13)	N4—C17—C18	115.7 (2)
O9—S2—C28	103.82 (13)	C16—C17—C18	121.5 (2)
C5—N1—C1	118.9 (2)	O3—C18—N5	127.5 (2)
C5—N1—Cu1	113.08 (17)	O3—C18—C17	121.2 (2)
C1—N1—Cu1	128.01 (18)	N5—C18—C17	111.3 (2)
C6—N2—C7	124.7 (2)	O4—C19—N5	128.4 (2)
C6—N2—Cu1	117.27 (17)	O4—C19—C20	118.8 (2)
C7—N2—Cu1	117.67 (17)	N5—C19—C20	112.7 (2)
C12—N3—C8	118.8 (2)	N6—C20—C21	123.2 (2)
C12—N3—Cu1	127.81 (18)	N6—C20—C19	115.0 (2)
C8—N3—Cu1	112.95 (17)	C21—C20—C19	121.8 (2)
C13—N4—C17	118.3 (2)	C20—C21—C22	118.1 (2)
C13—N4—Cu2	128.22 (19)	C20—C21—H21	120.9
C17—N4—Cu2	113.47 (17)	C22—C21—H21	120.9
C19—N5—C18	124.5 (2)	C23—C22—C21	118.8 (3)
C19—N5—Cu2	117.75 (17)	C23—C22—H22	120.6
C18—N5—Cu2	117.49 (17)	C21—C22—H22	120.6
C24—N6—C20	118.2 (2)	C22—C23—C24	119.7 (3)
C24—N6—Cu2	128.81 (19)	C22—C23—H23	120.2
C20—N6—Cu2	112.96 (17)	C24—C23—H23	120.2
C25—N7—Cu2	169.2 (2)	N6—C24—C23	122.0 (3)
C18—O3—Cu1	123.40 (17)	N6—C24—H24	119.0
C19—O4—Cu1	130.64 (17)	C23—C24—H24	119.0
S1—O5—Cu2	128.85 (12)	N7—C25—C26	178.2 (3)
N1—C1—C2	121.9 (3)	C25—C26—H26A	109.5
N1—C1—H1	119.0	C25—C26—H26B	109.5
C2—C1—H1	119.0	H26A—C26—H26B	109.5
C3—C2—C1	118.7 (3)	C25—C26—H26C	109.5
C3—C2—H2	120.6	H26A—C26—H26C	109.5
C1—C2—H2	120.6	H26B—C26—H26C	109.5
C2—C3—C4	119.8 (3)	F2—C27—F3	107.4 (3)
C2—C3—H3	120.1	F2—C27—F1	107.6 (3)
C4—C3—H3	120.1	F3—C27—F1	107.6 (3)
C3—C4—C5	118.5 (3)	F2—C27—S1	112.3 (2)
C3—C4—H4	120.7	F3—C27—S1	111.2 (2)
C5—C4—H4	120.7	F1—C27—S1	110.5 (2)
N1—C5—C4	122.2 (2)	F4—C28—F5	108.4 (2)
N1—C5—C6	116.2 (2)	F4—C28—F6	106.6 (2)
C4—C5—C6	121.6 (2)	F5—C28—F6	107.3 (2)
O1—C6—N2	128.9 (2)	F4—C28—S2	112.5 (2)
O1—C6—C5	121.0 (2)	F5—C28—S2	111.41 (19)
N2—C6—C5	110.1 (2)	F6—C28—S2	110.37 (19)
O2—C7—N2	129.0 (2)		
			
N2—Cu1—N1—C5	−3.42 (18)	N1—C5—C6—N2	4.8 (3)
O4—Cu1—N1—C5	−164.09 (17)	C4—C5—C6—N2	−176.5 (2)
N3—Cu1—N1—C5	−7.1 (4)	C6—N2—C7—O2	−7.3 (4)
O3—Cu1—N1—C5	110.98 (17)	Cu1—N2—C7—O2	179.8 (2)
N2—Cu1—N1—C1	179.3 (2)	C6—N2—C7—C8	172.0 (2)
O4—Cu1—N1—C1	18.6 (2)	Cu1—N2—C7—C8	−0.9 (3)
N3—Cu1—N1—C1	175.6 (3)	C12—N3—C8—C9	1.4 (4)
O3—Cu1—N1—C1	−66.4 (2)	Cu1—N3—C8—C9	174.49 (19)
O4—Cu1—N2—C6	88.9 (3)	C12—N3—C8—C7	−177.1 (2)
N1—Cu1—N2—C6	6.56 (18)	Cu1—N3—C8—C7	−4.1 (3)
N3—Cu1—N2—C6	−174.4 (2)	O2—C7—C8—N3	−177.3 (2)
O3—Cu1—N2—C6	−90.82 (19)	N2—C7—C8—N3	3.3 (3)
O4—Cu1—N2—C7	−97.7 (3)	O2—C7—C8—C9	4.1 (4)
N1—Cu1—N2—C7	179.96 (19)	N2—C7—C8—C9	−175.3 (2)
N3—Cu1—N2—C7	−0.96 (18)	N3—C8—C9—C10	−1.0 (4)
O3—Cu1—N2—C7	82.58 (19)	C7—C8—C9—C10	177.4 (2)
N2—Cu1—N3—C12	175.1 (2)	C8—C9—C10—C11	−0.5 (4)
O4—Cu1—N3—C12	−24.5 (2)	C9—C10—C11—C12	1.7 (4)
N1—Cu1—N3—C12	178.7 (3)	C8—N3—C12—C11	−0.2 (4)
O3—Cu1—N3—C12	59.3 (2)	Cu1—N3—C12—C11	−172.1 (2)
N2—Cu1—N3—C8	2.79 (17)	C10—C11—C12—N3	−1.3 (4)
O4—Cu1—N3—C8	163.21 (17)	C17—N4—C13—C14	−1.4 (4)
N1—Cu1—N3—C8	6.4 (4)	Cu2—N4—C13—C14	−178.7 (2)
O3—Cu1—N3—C8	−113.00 (17)	N4—C13—C14—C15	0.3 (4)
N5—Cu2—N4—C13	−179.7 (2)	C13—C14—C15—C16	0.9 (4)
N7—Cu2—N4—C13	−4.4 (2)	C14—C15—C16—C17	−1.0 (4)
N6—Cu2—N4—C13	171.5 (3)	C13—N4—C17—C16	1.3 (4)
O5—Cu2—N4—C13	79.6 (2)	Cu2—N4—C17—C16	179.0 (2)
N5—Cu2—N4—C17	2.86 (18)	C13—N4—C17—C18	−177.8 (2)
N7—Cu2—N4—C17	178.15 (18)	Cu2—N4—C17—C18	−0.1 (3)
N6—Cu2—N4—C17	−6.0 (4)	C15—C16—C17—N4	−0.1 (4)
O5—Cu2—N4—C17	−97.92 (18)	C15—C16—C17—C18	178.9 (2)
N4—Cu2—N5—C19	−179.5 (2)	Cu1—O3—C18—N5	−10.9 (4)
N6—Cu2—N5—C19	−2.17 (19)	Cu1—O3—C18—C17	169.94 (17)
O5—Cu2—N5—C19	−97.01 (19)	C19—N5—C18—O3	1.1 (4)
N4—Cu2—N5—C18	−5.51 (19)	Cu2—N5—C18—O3	−172.5 (2)
N6—Cu2—N5—C18	171.9 (2)	C19—N5—C18—C17	−179.7 (2)
O5—Cu2—N5—C18	77.03 (19)	Cu2—N5—C18—C17	6.7 (3)
N5—Cu2—N6—C24	−179.7 (2)	N4—C17—C18—O3	175.1 (2)
N7—Cu2—N6—C24	5.0 (2)	C16—C17—C18—O3	−4.0 (4)
N4—Cu2—N6—C24	−170.9 (3)	N4—C17—C18—N5	−4.2 (3)
O5—Cu2—N6—C24	−80.8 (2)	C16—C17—C18—N5	176.7 (2)
N5—Cu2—N6—C20	2.40 (17)	Cu1—O4—C19—N5	−6.8 (4)
N7—Cu2—N6—C20	−172.86 (18)	Cu1—O4—C19—C20	174.63 (16)
N4—Cu2—N6—C20	11.3 (4)	C18—N5—C19—O4	9.3 (4)
O5—Cu2—N6—C20	101.37 (17)	Cu2—N5—C19—O4	−177.1 (2)
N4—Cu2—N7—C25	−158.9 (13)	C18—N5—C19—C20	−172.1 (2)
N6—Cu2—N7—C25	22.3 (13)	Cu2—N5—C19—C20	1.5 (3)
O5—Cu2—N7—C25	117.9 (13)	C24—N6—C20—C21	−0.1 (4)
N2—Cu1—O3—C18	−170.46 (19)	Cu2—N6—C20—C21	178.0 (2)
O4—Cu1—O3—C18	9.6 (2)	C24—N6—C20—C19	179.6 (2)
N1—Cu1—O3—C18	103.0 (2)	Cu2—N6—C20—C19	−2.3 (3)
N3—Cu1—O3—C18	−89.9 (2)	O4—C19—C20—N6	179.4 (2)
N2—Cu1—O4—C19	178.8 (2)	N5—C19—C20—N6	0.6 (3)
N1—Cu1—O4—C19	−100.9 (2)	O4—C19—C20—C21	−0.9 (4)
N3—Cu1—O4—C19	84.8 (2)	N5—C19—C20—C21	−179.7 (2)
O3—Cu1—O4—C19	−1.5 (2)	N6—C20—C21—C22	−0.1 (4)
O6—S1—O5—Cu2	130.69 (15)	C19—C20—C21—C22	−179.8 (2)
O7—S1—O5—Cu2	−6.5 (2)	C20—C21—C22—C23	0.1 (4)
C27—S1—O5—Cu2	−117.89 (17)	C21—C22—C23—C24	0.0 (4)
N5—Cu2—O5—S1	22.78 (18)	C20—N6—C24—C23	0.2 (4)
N7—Cu2—O5—S1	−155.98 (17)	Cu2—N6—C24—C23	−177.5 (2)
N4—Cu2—O5—S1	103.22 (17)	C22—C23—C24—N6	−0.2 (4)
N6—Cu2—O5—S1	−59.61 (17)	O5—S1—C27—F2	−177.7 (2)
C5—N1—C1—C2	1.0 (4)	O6—S1—C27—F2	−57.9 (3)
Cu1—N1—C1—C2	178.2 (2)	O7—S1—C27—F2	62.9 (3)
N1—C1—C2—C3	−0.5 (4)	O5—S1—C27—F3	61.9 (3)
C1—C2—C3—C4	−0.2 (4)	O6—S1—C27—F3	−178.2 (2)
C2—C3—C4—C5	0.2 (4)	O7—S1—C27—F3	−57.5 (3)
C1—N1—C5—C4	−0.9 (4)	O5—S1—C27—F1	−57.5 (3)
Cu1—N1—C5—C4	−178.5 (2)	O6—S1—C27—F1	62.3 (3)
C1—N1—C5—C6	177.7 (2)	O7—S1—C27—F1	−176.9 (2)
Cu1—N1—C5—C6	0.1 (3)	O8—S2—C28—F4	−47.3 (2)
C3—C4—C5—N1	0.3 (4)	O10—S2—C28—F4	−168.5 (2)
C3—C4—C5—C6	−178.3 (2)	O9—S2—C28—F4	72.8 (2)
C7—N2—C6—O1	−2.7 (4)	O8—S2—C28—F5	−169.3 (2)
Cu1—N2—C6—O1	170.2 (2)	O10—S2—C28—F5	69.5 (2)
C7—N2—C6—C5	179.3 (2)	O9—S2—C28—F5	−49.2 (2)
Cu1—N2—C6—C5	−7.8 (3)	O8—S2—C28—F6	71.6 (2)
N1—C5—C6—O1	−173.4 (2)	O10—S2—C28—F6	−49.6 (2)
C4—C5—C6—O1	5.3 (4)	O9—S2—C28—F6	−168.27 (19)

### Hydrogen-bond geometry (Å, º)

**Table:**

*D*—H···*A*	*D*—H	H···*A*	*D*···*A*	*D*—H···*A*
C2—H2···O9^i^	0.95	2.57	3.448 (3)	154
C4—H4···O1^ii^	0.95	2.53	3.210 (4)	128
C10—H10···F5^iii^	0.95	2.54	3.239 (3)	130
C21—H21···O1^iv^	0.95	2.48	3.257 (3)	140
C21—H21···O2^iv^	0.95	2.37	3.203 (3)	147
C22—H22···O10^v^	0.95	2.48	3.400 (3)	163

Symmetry codes: (i) *x*+1, *y*−1, *z*; (ii) −*x*+2, −*y*, −*z*+1; (iii) −*x*+1, −*y*+2, −*z*+1; (iv) −*x*+2, −*y*+1, −*z*+1; (v) *x*+1, *y*, *z*.
